# Integrative analysis of the 3D genome structure reveals that CTCF maintains the properties of mouse female germline stem cells

**DOI:** 10.1007/s00018-021-04107-y

**Published:** 2022-01-03

**Authors:** Geng G. Tian, Xinyan Zhao, Changliang Hou, Wenhai Xie, Xiaoyong Li, Yinjuan Wang, Lijuan Wang, Hua Li, Xiaodong Zhao, Jing Li, Ji Wu

**Affiliations:** 1 Renji Hospital, Key Laboratory for the Genetics of Developmental and Neuropsychiatric Disorders (Ministry of Education), Bio-X Institutes, School of Medicine, Shanghai Jiao Tong University, Shanghai, 200240 China; 2 Key Laboratory of Fertility Preservation and Maintenance of Ministry of Education, Ningxia Medical University, Yinchuan, 750004 China; 3 Westlake Laboratory of Life Sciences and Biomedicine, School of Life Sciences, Westlake University, Hangzhou, 310024 Zhejiang China; 4 Key Laboratory of Bioorganic Synthesis of Zhejiang Province, College of Biotechnology and Bioengineering, Zhejiang University of Technology, Hangzhou, 310014 China; 5 State Key Laboratory for Oncogenes and Bio-ID Center, School of Biomedical Engineering, Shanghai Jiao Tong University, 800 Dongchuan Road, Shanghai, 200240 China; 6 Shanghai Center for Systems Biomedicine, Shanghai Jiao Tong University, Shanghai, 200240 China; 7 Department of Bioinformatics and Biostatistics, School of Life Sciences and Biotechnology, Shanghai Jiao Tong University, 800 Dongchuan Road, Shanghai, 200240 China

**Keywords:** Stem cell, Chromatin structure, Hi-C, CTCF

## Abstract

The three-dimensional configuration of the genome ensures cell type-specific gene expression profiles by placing genes and regulatory elements in close spatial proximity. Here, we used in situ high-throughput chromosome conformation (in situ Hi-C), RNA sequencing (RNA-seq) and chromatin immunoprecipitation sequencing (ChIP-seq) to characterize the high-order chromatin structure signature of female germline stem cells (FGSCs) and identify its regulating key factor based on the data-driven of multiple omics data. By comparison with pluripotent stem cells (PSCs), adult stem cells (ASCs), and somatic cells at three major levels of chromatin architecture, A/B compartments, topologically associating domains, and chromatin loops, the chromatin architecture of FGSCs was most similar to that of other ASCs and largely different from that of PSCs and somatic cells. After integrative analysis of the three-dimensional chromatin structure, active compartment-associating loops (aCALs) were identified as a signature of high-order chromatin organization in FGSCs, which revealed that CCCTC-binding factor was a major factor to maintain the properties of FGSCs through regulation of aCALs. We found FGSCs belong to ASCs at chromatin structure level and characterized aCALs as the high-order chromatin structure signature of FGSCs. Furthermore, CTCF was identified to play a key role in regulating aCALS to maintain the biological functions of FGSCs. These data provide a valuable resource for future studies of the features of chromatin organization in mammalian stem cells and further understanding of the fundamental characteristics of FGSCs.

**Supplementary Information:**

The online version contains supplementary material available at 10.1007/s00018-021-04107-y.

## Introduction

The chromatin architecture of germline stem cells (GSCs) carries the information necessary for cells to perform their unique functions and is thus an essential factor in the transmission of the genome from generation to generation. GSCs renew themselves and differentiate into gametes that include sperm and metaphase II (MII) oocytes [1–3]. During this process, spermatogonial stem cells (SSCs) differentiate into sperm by packaging chromatin into a highly condensed configuration. Recently identified female GSCs (FGSCs) in postnatal ovaries differentiate into MII oocytes after transplantation into the ovaries of infertile mice [2–8]; thereby, reshaping the concept that female mammals lose their ability to produce oocytes at birth [9, 10]. Unlike other stem cells, GSCs undergo meiosis to produce haploid gametes with chromatin remodeling. It is therefore necessary to characterize the chromatin structure of GSCs during their development to further understand GSC biology.

High-throughput chromosome conformation (Hi-C) is a powerful technology to study genome-wide architecture, which allows the high-order chromatin structure to be displayed and reveals the chromatin organization in the nucleus [11]. The spatial organization of chromatin as the structural and functional basis of the genome affects DNA localization with important roles in regulation of gene expression to ensure DNA duplication and other biological processes [12, 13], which suggests a close connection to the biological functions and signature of the cell. Dixon et al. found extensive chromatin reorganization during specification of human embryonic stem (ES) cell-derived lineages by mapping genome-wide chromatin interactions in human ES cells and four human ES cell-derived lineages [14]. Previous studies have reported that the chromatin architecture changes dynamically during spermatogenesis with dissolved and reappeared topologically associating domains (TADs) and A/B compartments [15, 16]. The high-order chromatin organization exhibits statistically significant differences between sperm and embryonic fibroblasts [17, 18]. However, the signature of the chromatin architecture of FGSCs is unknown.

CCCTC-binding factor (CTCF) is a critical regulator of chromatin architecture, which underlies its functions that include gene expression and three-dimensional (3D) genome construction and regulation [19–22]. In mammals, CTCF recognizes chromatin insulators to segment chromatin into several TADs. Therefore, disruption of CTCF expression can led to the disappearance of TAD formation [23]. Notably, CTCF regulates gene expression through the formation of chromatin loops [19, 20]. A recent study has reported that CTCF plays an important role in establishment of the 3D chromatin structure during human embryogenesis, which indicates that CTCF is also important for development [24]. However, the 3D architectural role of CTCF in FGSCs is unknown.

In this study, we used in situ Hi-C technology to compare the chromatin organizations of FGSCs and induced pluripotent stem cells (iPSCs), adult stem cells (ASCs), which included SSCs and neural stem cells (NSCs), and somatic cells (mouse SIM embryonic fibroblasts; STO cells) to explore the chromosome structure characteristics of FGSCs. Together with RNA sequencing (RNA-seq) and chromatin immunoprecipitation sequencing (ChIP-seq), we identified distinct features (active compartment-associating loops; aCALs) of the chromatin organization in FGSCs at three major levels: A/B compartments, TADs, and chromatin loops, and found that FGSCs were most similar to other ASCs and largely different from iPSCs and STO cells. By further analysis of the distinct features, we revealed that CTCF may be a crucial factor to maintain the properties of FGSCs through regulation of the high-order chromatin structure.

## Materials and methods

### Animals

The CAG-EGFP mice from the Model Animal Research Center of Nanjing University, also named C57BL/6-Tg (CAG-EGFP) C14-Y01-FM131-Osb [25]. The Ddx4-Cre; mT/mG mice were generated as described previously [26]. DBA/2 mice were obtained from the Shanghai Slac Laboratory Animal Co., Ltd. (Shanghai, China). All procedures involving animals were approved by the Institutional Animal Care and Use Committee (IACUC) at Shanghai Jiao Tong University, and all experiments were performed in accordance with the approved protocols.

### Isolation and culture of SSCs

Mouse SSCs were isolated and cultured from 6-day-old male F_1_ progeny of DBA/2 × CAG-EGFP mice, as previously described [27]. In brief, SSCs (> 20 passages) were cultured on mitomycin C (MMC)-treated mouse embryonic fibroblast (MEF) feeder cells with SSC culture medium. The SSC culture medium included StemPro-34 SFM supplemented with StemPro supplement (Invitrogen, Carlsbad, CA, USA), human basic fibroblast growth factor (100 μg/ml transferrin, 25 μg/ml insulin, 10 ng/ml; Invitrogen), recombinant human epidermal growth factor (20 ng/ml; Invitrogen), recombinant human glial cell line-derived neurotrophic factor (10 ng/ml; Invitrogen), d-(+)-glucose (6 mg/ml; Invitrogen), putrescine (60 mM; Invitrogen), sodium selenite (30 nM; Invitrogen), l-glutamine (2 mM; Invitrogen), pyruvic acid (30 μg/ml), dl-lactic acid (1 μl/ml; Sigma, St. Louis, MO, USA), bovine serum albumin (5 mg/ml; Sigma), 2-mercaptoethanol (10 μM; Sigma), 1× MEM vitamin solution (Invitrogen), 1× nonessential amino acid solution (Invitrogen), ascorbic acid (0.1 mM), d-biotin (10 μg/ml; Sigma), 1% fetal bovine serum (Gibco), and 1× penicillin/streptomycin solution (Invitrogen). The medium was changed every 2–3 days.

### Isolation and culture of FGSCs

Mouse FGSCs were isolated and cultured from neonatal Ddx4-Cre; mT/mG mouse ovaries (6 days old), as previously described [26]. Briefly, FGSCs (> 18 passages) were cultured on mitotically inactivated STO (SIM mouse embryo-derived thioguanine- and ouabain-resistant) cell feeders in minimum essential medium alpha (MEMα; Invitrogen) supplemented with 10% fetal bovine serum (FBS) (Life Technologies), mouse leukemia inhibitory factor (10 ng/ml; Santa Cruz Biotechnology), mouse epidermal growth factor (EGF) (20 ng/ml; PeproTech), basic fibroblast growth factor (bFGF) (10 ng/ml; PeproTech), mouse glial cell line-derived neurotrophic factor (GDNF) (10 ng/ml; PeproTech), nonessential amino acids (1 mM; Life Technologies), l-glutamine (2 mM; Sigma), pyruvate (30 mg/ml; Amresco), and β-mercaptoethanol (50 mM; Biotech). The FGSCs were subcultured every 4–7 days.

### Isolation and culture of NSCs

NSCs were isolated from E12.5 mouse embryonic cortex, in accordance with a previously described procedure [28, 29]. The primary NSCs were seeded onto poly-l-ornithine (Sigma-Aldrich)- and laminin (Invitrogen)-coated dishes, cultured as monocultures. Neural basal medium with EGF (20 ng/ml; PeproTech), bFGF (20 ng/ml; PeproTech), heparin (20 ng/ml; Sigma-Aldrich), and 2% B27 (Invitrogen) was used as NSC proliferation medium. For differentiation, NSCs were seeded on poly-L-ornithine (Sigma-Aldrich)- and laminin (Invitrogen)-coated dishes. Upon NSC attachment, the medium was changed to differentiation medium identical to the NSC proliferation medium without growth factors (EGF and bFGF). After differentiation for 10 days, neural- and glial-specific markers were used to determine the differentiation potential of cultured NSCs.

### STO culture

STOs were maintained in Dulbecco’s modified Eagle’s medium (DMEM) with high glucose (Life Technologies), 10% FBS (Life Technologies), 1% nonessential amino acids (Life Technologies), glutamine (2 mM; Sigma), and penicillin (100 U/ml; Sigma)/streptomycin (0.1 mg/ml; Sigma) at 37 °C and 5% CO_2_, and passaged after 3–4 days.

### Knockdown of CTCF in FGSCs

To examine the function of CTCF in mouse FGSCs, the following shRNAs targeting CTCF were designed and synthesized by OBiO Technology (Shanghai, China) Corp., Ltd (Suppl. Table S5). Then, the sequence with the best interference effect was used for lentivirus packaging. The sequences of shRNA were *CTCF*: 5′-GCGAAAGCAGCATTCCTAT-3′. For lentivirus infection, 60–70% cell on 48-well plate incubated with 1:1 mixture of culture medium and lentivirus solution. After overnight infection, we changed fresh culture medium and cultured for 12 h. To select for positive cells, 100 ng/ml puromycin was added to the FGSC culture medium for 72 h. The surviving FGSCs cells were collected and analyzed by qRT-PCR.

### Immunofluorescence

The immunofluorescence procedure was performed as described previously with minor modification [30]. Cultured cells were washed twice with phosphate-buffered saline (PBS) and then fixed in 4% paraformaldehyde for 15 min. After that the cells were rinsed twice and incubated with PBS containing 0.1% (v/v) Triton X-100 for 15 min. Fixed cells were blocked by 10% normal horse serum in PBS for 10 min at room temperature. The cells were then incubated with primary antibodies: mouse monoclonal anti-NESTIN and rabbit polyclonal anti-SOX2 (1:180 dilution; Abcam), mouse polyclonal anti-TUJ-1 and rabbit polyclonal anti-GFAP (1:150 dilution; Abcam), mouse monoclonal anti-MVH (1:500 dilution; Abcam), mouse monoclonal anti-PLZF (1:100 dilution; Santa Cruz Biotechnology). After 1 h of incubation at room temperature with the primary antibodies, the cells were rinsed twice in PBS and then incubated in the dark with a fluorescein isothiocyanate-conjugated secondary antibody, either goat antirabbit IgG or goat anti-mouse IgG (1:200 dilution; Proteintech, Chicago, IL, USA) for 60 min at 37 °C. This was followed by rinsing and staining of the nucleus with 4′,6-diamidino-2-phenylindole (DAPI) (1:1000 dilution; Sigma)-containing PBS for 10 min at room temperature. After washing twice with PBS, the cells were examined in fresh PBS under an inverted fluorescence microscope (Leica).

### Western blot assay

Protein extracts were separated by 7–12% SDS–polyacrylamide gel electrophoresis (PAGE), and then transferred to 0.45 μm PVDF membranes. The membrane was blocked in 5% TBST milk for 1 h and probed overnight with antibodies against CTCF (1:3000, Sigma-Aldrich). The membrane was thrice washed in 0.1% TBS-Tween-20, incubated with diluted secondary antibodies (Proteintech) in 5% PBST milk for 2 h and the antigen–antibody reaction was visualized by enhanced chemiluminescence assay (ECL, Thermo). Western blot quantification was performed with ImageJ software.

### Cell proliferation assay

Cell proliferation rates was assayed using the cell counting kit-8 (Genomeditech, Shanghai, China) and Cell-Light EdU Apollo^®^567 in Vitro Imaging Kit (RiboBio, Guangzhou, China) according to the manufacturer’s instructions. In brief, for CCK8 assay, 10 μl CCK8 solution was added to each well containing 100 μl medium. The plate was incubated for additional 4 h before measuring the absorbance at 450 nm wavelength. For EdU labeling assay, the 5-ethynyl-2′-deoxyuridine (EdU) was added to each well for 2 h, and the cells were fixed and stained by 1× Apollo for 30 min. Then, the cell nucleus was counterstained with 1× Hoechst 33342. The stained cells were examined with Leica fluorescence microscope and photographed with camera.

### RT-PCR and qRT-PCR

Total RNA from cells was extracted using Trizol reagent (Invitrogen), in accordance with the manufacturer’s instructions. After extraction, total RNA (1 µg) was used to synthesize cDNA with Primescript Reverse Transcriptase (ThermoFisher). qRT-PCR analysis was carried out with SYBR Premix Ex Taq (YEASEN, Shanghai, China) in a 20ul volume on an Applied Biosystems 7500 Real-Time PCR System. Specific marker genes, such as *Mvh*, *Oct4*, *Fragilis*, *Stella*, *Dazl*, *Exv5*, *Plzf*, *Gfra1*,* Nestin*, *Sox2*, *Pax6*, *Olig2*, *Ascl1*, *Gfap*, *Meikin*,* Prdm9*,* Hspa1b*,* Majin,* and *Notch2*, were used to characterize the cells. *Gapdh* was used as an internal control. Primer sequences are listed in Supplementary Table S1.

### In situ Hi-C library generation

Cells were used for in situ Hi-C, including iPSCs (from Kang’s Lab, Tongji University), FGSCs (collection with fluorescence activated cell sorting, FACS), SSCs, NSCs, and STOs [31]. Hi-C libraries were generated in accordance with the standard in situ Hi-C protocol, with minor modification [19]. Five million cells were harvested with 0.05% trypsin, washed twice, resuspended with DMEM medium and then crosslinked with 37% formaldehyde (F8775; Sigma) to a final concentration of 1% for 10 min at room temperature. Formaldehyde was quenched by adding glycine to a final concentration of 0.2 M and the cells were incubated for 5 min at room temperature, then transferred to ice for 20 min. The fixed cells were centrifuged at 400×*g* and 4 °C and washed with cold PBS once, followed by storage at − 80 °C. Fixed cells were resuspended in lysis buffer and then incubated on ice for 30 min. Nuclei were pelleted by centrifugation at 3000×*g* for 5 min and washed once with 500 μl of cold lysis buffer. The pellet was resuspended in 0.5% sodium dodecyl sulfate (SDS) and incubated at 62 °C for 5–10 min. Then, SDS was quenched by adding water (145 μl) and 10% Triton X-100 (25 μl) and incubated at 37 °C for 15 min. The nuclei were digested overnight at 37 °C with 100U Mbol restriction enzyme (20 μl; NEB, R0417) and 10× NEBuffer 2 (25 μl). The next day, the nuclei were incubated at 62 °C for 20 min, followed by cooling to room temperature. A mixed solution (50 μl) (37.5 μl of 0.4 mM biotin-14-dATP, 1.5 μl of 10 mM dCTP, 1.5 μl of 10 mM dGTP, 1.5 μl of 10 mM dTTP, and 8 μl of 5 U/μl DNA Polymerase I) was then added and incubated at 37 °C for 2 h. Ligation master mix (663 μl of water, 120 μl of 10× NEB T4 DNA ligase buffer, 100 μl of 10% Triton X-100, 12 μl of 10 mg/ml bovine serum albumin, and 5 μl of 400 U/μl T4 DNA ligase) was added to a volume of 900 μl and slowly rotated for 6 h at room temperature. Next, to degrade protein, proteinase K (50 μl of final concentration 20 mg/ml) and 10% SDS (120 μl) were added and incubated at 55 °C for 30 min. Then, sodium chloride (5 M, 130 μl) was added and incubated at 68 °C overnight. On the third day, the DNA was purified by adding 1.6× volumes of pure ethanol and 0.1× volumes of sodium acetate (3 M), pH 5.2. Subsequently, DNA was diluted with Tris buffer and sheared by a Digital Sonifier (Branson). Dynabeads MyOne Streptavidin T1 beads (50 μl; Thermo Scientific) were added and washed once with 1× Tween washing buffer (TWB) (5 mM Tris–HCl (pH 7.5), 0.5 mM EDTA, 1 M NaCl, 0.05 Tween 20), separated on a magnet, and then the solution was discarded. The beads were resuspended in 2× binding buffer (10 mM Tris–HCl (pH 7.5), 1 mM EDTA, 2 M NaCl), mixed with the DNA, and incubated at room temperature for 1 h with rotation, followed by washing twice with TWB (500 μl) and reclaiming the beads using a magnet. The beads were next resuspended with a mixture [88 μl of 1× NEB T4 DNA ligase buffer with 10 mM ATP, 2 μl of 25 mM dNTP mix, 5 μl of 10 U/μl NEB T4 PNK, 4 μl of 3 U/μl NEB T4 DNA polymerase I, 1 μl of 5 U/μl NEB DNA polymerase I, Large (Klenow) Fragment] and incubated at room temperature for 30 min, followed by separation on a magnet and washing twice with 1× TWB. The beads were resuspended with mixture (90 μl of 1× NEBuffer 2, 5 μl of 10 mM dATP, 5 μl of 5 U/μl NEB KlenowExo Minus) and incubated at 37 °C for 30 min. Then, separation on a magnet was performed, after which the solution was discarded and washing was carried out twice. The beads were resuspended in 1× NEB Quick ligation reaction buffer (50 μl; NEB), and NEB DNA Quick ligase (2 μl; NEB) and an Illumina indexed adapter of NEBNext Multiplex Oligos for Illumina (3 μl; NEB) were added at room temperature for 15 min. Then, USER enzyme of NEBNext Multiplex Oligos for Illumina (3 μl) was added to the ligation mixture at 37 °C for 15 min. The DNA was washed twice and eluted by resuspending the beads with Tris–HCl (50 μl of 10 mM; pH 8.0), followed by incubation at 98 °C for 10 min. The DNA suspension was transferred into a fresh tube and stored at − 20 °C. The Hi-C library was amplified in a PCR system (25 μl of NEBNext Q5 Hot Start HiFi PCR mix, 5 μl of index primer of NEBNext Multiplex Oligos for Illumina, 5 μl of universal primer of NEBNext Multiplex Oligos for Illumina, and 15 μl of DNA template) with the following PCR conditions: initial denaturation at 98 °C for 30 s; eight cycles of denaturation at 98 °C for 10 s and annealing/extension at 65 °C for 75 s; followed by final extension at 65 °C for 5 min, and then maintenance at 4 °C. The DNA fraction in the size range of 300–500 bp was selected using Agencourt AMPure XP beads (Beckman Coulter). The DNA was eluted with 1× Tris–HCl buffer (33 μl) and incubated at room temperature for 5 min, followed by separation on a magnet and transfer of the solution to a fresh labeled tube. This produced the final Hi-C library. The quality of the Hi-C library was determined using the Qubit dsDNA HS Assay and Agilent 2100 DNA 1000 HS kit. The high-quality libraries were sequenced using an Illumina sequencing platform.

### ChIP-Seq library preparation

The preparation of ChIP and input DNA libraries was performed as previously described [32]. In brief, two cells were crosslinked with 1% formaldehyde for 5 min at room temperature and quenched with glycine (125 mM). Cells were then put on ice, resuspended in cold cell lysis buffer [140 mM NaCl, 1 mM EDTA pH 8.0, 1% Triton X-100, 0.1% SDS, and protease inhibitors (Roche)]. Nuclei were sonicated into fragments of 200–1000 bp in size. The chromatin fragments were precleared and then immunoprecipitated with Protein A + G magnetic beads coupled with anti-H3K4me3 (ab8580; Abcam), anti-H3K27ac (ab4729; Abcam), anti-H3K27me3 (07-449; Millipore), and anti-CTCF (ab70303; Abcam). After reverse crosslinking, immunoprecipitated DNA and input DNA were end-repaired and adapters were ligated to the DNA fragments using NEBNext Ultra End-Repair/dA-Tailing Module (E7442; NEB) and NEBNext Ultra Ligation Module (E7445; NEB). High-throughput sequencing of the ChIP fragments was performed using Illumina NextSeq 500, following the manufacturer’s protocol.

### Hi-C data processing, mapping, and ICE normalization

Hi-C pair-end was trimmed of adaptor sequences and low-quality reads were filtered with BBmap (version 38.16). HiCPro (version 2.7) [33] was then used to map, process, and perform iterative correction for normalization. Reads were independently aligned to the mouse reference genome (mm9) with the bowtie2 algorithm [34]. Uncut DNA reads, re-ligation reads, continuous reads, and PCR artifacts were discarded. We then constructed a contact matrix using the unique mapped reads (MAPQ > 10). We divided the genome into sequential bins of equal size and valid read pairs were then binned at a specific resolution. ICE [35] normalization was applied to remove bias in the raw matrix, such as GC content, mappability, and effective fragment length in Hi-C data. Contact matrices were finally generated at binning resolutions of 10, 20, 40, 200, and 400 kb.

### Validation of Hi-C data

The data reproducibility was confirmed by calculating Pearson’s correlation coefficient between the two Hi-C repeats. For each possible interaction *I*_*ij*_ between two replicates, these were correlated by comparing each point’s interaction in the normalized interaction matrix. Considering that the interaction matrix was highly skewed toward proximal interactions, we calculated the correlation to a maximum distance restricted to 2 Mb between points *i* and *j*. R was used to calculate Pearson’s correlation between two duplicates.

### Contact probability *p*(*s*) calculation

*p*(*s*) only considering intra interactions, was calculated with normalized interaction matrices at 40-kb resolution, as described previously [36]. Briefly, we divided the genome into 40-kb bins and counted the number of interactions at corresponding distances for each distance (separated by 40, 80, 120, 160 kb, etc.). We then divided the number of interactions in each bin by the total number of possible region reads as *p*(*s*). Finally, we normalized the sum of *p*(*s*) over the range of distances as 1. The curve (log–log axis) was generated by locally weighted scatterplot smoothing.

### Identification of A and B compartments

The R package (HiTC) [37] was used to generate the PC1 eigenvectors using 400-kb normalized matrices with pca.hic function, using the options: (normPerExpected = TRUE, npc = 1), for which a positive value indicates the A compartment and a negative value indicated the B compartment. To investigate compartment switching, we defined switched bins only if the PC1 eigenvectors changed in the same direction for two replicates.

### Identification of concordant genes with A/B compartment switch

We defined genes with concordant changes in expression and compartment status according to a previously described method, with minor modifications [14]. Briefly, the covariance between the vector of the gene expression values (FPKM) and the vector of PC1 values for each gene was calculated across five cell types. We then used the covariance metric to quantitatively define ‘concordance’. The observed covariance values were compared with a random background distribution to calculate a *p* value for the covariance for each gene. Randomly shuffling the vector of FPKM for each gene produced the background distribution, and then obtained the covariance between the PC1 values and the random gene expression vector. A rank-based *p* value could be calculated for the observed covariance values with 1000 repeats for each gene. Concordant genes were defined as those with a *p* value < 0.01.

### TAD calling and TAD boundaries

We calculated the location of the TADs using the directional index (DI) value, as previously described [38]. We first calculated the DI value for each bin based on the ICE-normalized and depth-normalized matrix and then used this value as the input for a hidden Markov model (HMM) to call TADs. TAD boundaries were defined as those < 400 kb.

### TAD signal calculation

We calculated TADs signals using the insulation score [39]. The insulation score for each bin in the 20 kb was calculated by the average number of interactions that occurred across each bin. Using this matrix, we then plotted the insulation score distribution centered in the FGSC TADs (up/down to 0.5 TAD).

### Identification of chromatin loops and calculation of APA score

We carried out chromatin loop calling using the tool (Juicer), as described previously [19]. The merged loops at different resolution as the calling chromatin loops. The calculation of APA score was followed the parameters: − *r* 25,000, 1000.

### ChIP-Seq data analysis

We aligned fastq files to the mm9 reference genome, removed PCR duplicates using Samtools (version 2.0.1) [40], and generated normalized genome coverage tracks from uniquely mapping reads (MAPQ > 10) using deepTools2 (version 3.1) [41]. Biological replicates were pooled, and coverage was then calculated as the average reads per million mapped reads (RPM) in 1-kb bins. To identify the correlation between ChIP-Seq and A/B compartment, we summed the log2 of the fold enrichment (treatment/input) from ChIP-Seq to calculate the relative ChIP-Seq signal in each compartment.

### RNA-Seq library generation and data analysis

Total RNA was extracted from 2 to 6 million cells using Trizol Reagent (Invitrogen). The RNA quality was assessed using Agilent Bioanalyzer 2100. RNA-Seq libraries were prepared using the KAPA Stranded mRNA-Seq kit, following the manufacturer’s instructions. After preparation, libraries were quantified using a Qubit fluorometer and sequenced with HiSeq Platform (2 × 150 bp). All RNA-Seq data were trimmed and aligned to the mm9 reference genome using Hisat2 (version 4.8.2) [42] with the default parameters. Gene expression FPKM was calculated by Cufflinks (version 2.2.1) [43] using the RefSeq database from the UCSC genome browser. Sequencing depth was normalized.

### GO term enrichment analysis

GO term enrichment analysis was performed using the DAVID tool (version 6.8) [44], with a focus on enriched biological processes (BP). The GO results were displayed by Cytoscape (version 3.5.1) [45]. For the Benjamin-corrected *p* value, a threshold of less than 0.05 was used for significance.

## Results

### Biological characterization of FGSCs and other ASCs

**Fig. 1:**
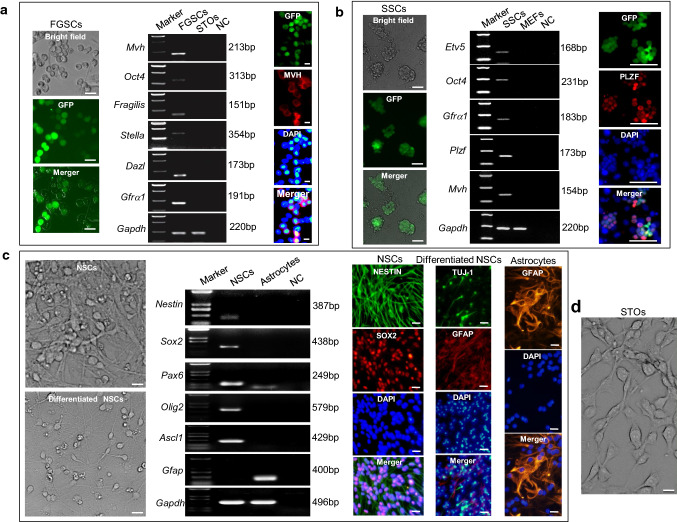
Morphology and biological characteristics of FGSCs and other ASCs. **a** Morphology and biological characteristics of cultured FGSCs (> 18 passages) from Ddx4-Cre; mT/mG mice. *Marker* 100-bp DNA marker. *NC* negative control with water. Scale bar, 25 µm (left) and 10 µm (right). **b** Morphology and biological characteristics of cultured SSCs (> 20 passages) from CAG-EGFP mice. *Marker* 100-bp DNA marker. *NC* is negative control with water. Scale bar, 25 µm (left) and 50 µm (right). **c** Morphology and biological characteristics of cultured NSCs and astrocytes. *Marker*100-bp DNA marker. *NC* is negative control with water. Scale bar, 10 µm. **d** Representative morphology of cultured STO cells. Scale bar, 10 µm

FGSCs were isolated from the ovaries of Ddx4-Cre;mT/mG neonatal mice and cultured as described previously [26]. After culture for at least 18 passages, the cells exhibited a characteristic morphology similar to that previously described for FGSCs [2, 26]. The expression of female germline marker genes was determined by reverse transcription-polymerase chain reaction (RT-PCR). FGSCs after long-term culture expressed *Oct4*, *Fragilis*, *Mvh* (mouse vasa homologue expressed exclusively in germ cells), *Stella*, *Gfrα1*, and *Dazl* genes. Furthermore, immunofluorescence analysis revealed that these cells expressed MVH, which confirmed their identity as FGSCs (Fig. 1a).

We isolated SSCs from the testes of 6-day-old DBA/2× CAG-EGFP F_1_ mice and cultured them as described previously [27]. Long-term cultured SSCs (> 20 passages) were assessed by RT-PCR and found to express male germline marker genes [*Etv5*, *Oct4*, *Plzf* (promyelocytic leukemia zinc finger), *Gfrα1*, and *Mvh*]. These results were confirmed by immunofluorescence and most cultured cells were also positive for PLZF expression, which confirmed their identity as SSCs (Fig. 1b).

Isolated primary NSCs self-proliferated and were cultured for 5–8 passages in NSC proliferation medium. Morphologically, cultured NSCs were shaped as spindles with a high nucleus-to-cytoplasm ratio as reported previously [29]. The cultured NSCs were positive for several NSC-specific markers, which included *Nestin*, *Sox2*, *Pax6*, *Olig2*, and *Ascl1*, as determined by RT-PCR. *Gfap* is a the standard marker for astrocytes as a negative control [46]. Immunocytochemical staining confirmed that most cultured NSCs were positive for NESTIN and SOX2 as typical markers specific for NSCs. Following the removal of epidermal growth factor and basic fibroblast growth factor from the medium, NSCs differentiated spontaneously into neurons and astrocytes as characterized by prominent dendrites with long axons and extensive cytoplasm with thick processes, respectively. The differentiation potential of cultured NSCs was confirmed by immunochemical staining of neural- and astrocyte-specific markers TUJ-1 (β3 tubulin) and GFAP (glial fibrillary acidic protein), respectively. These results confirmed the identity of the cultured cells as NSCs (Fig. 1c). The morphology of STO cells is shown in Fig. 1d.

### Global chromosome organization map of FGSCs

To reveal the signature of the chromatin architecture in FGSCs, we performed in situ Hi-C [19] with two biological replicates of FGSCs and other cells (SSCs, NSCs, iPSCs, and STO cells), which generated approximately 400 million reads for each replicate. After filtering artificial reads and normalization, we obtained 2 billion valid Hi-C reads over the five cell lines, which included an average of 207 million long range intrachromosomal *cis*-contacts and 90 million interchromosomal trans-contacts (Suppl. Table S1). We confirmed high reproducibility of the Hi-C data (Suppl. Figure S1) and combined the two biological replicates into a single set of merged Hi-C data per cell type to reach a maximum resolution of 20 kb.

**Fig. 2:**
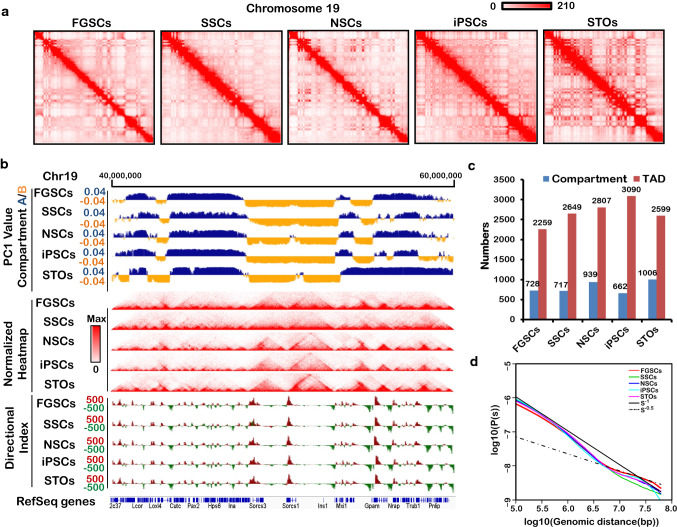
Overall chromosome structure in FGSCs. **a** Contact matrices of chromosome 19 in FGSCs, SSCs, NSCs, iPSCs, and STO cells. **b** First principal component (PC1) value, normalized Hi-C interaction heat maps, and directional indexes (DIs) of FGSCs and other cells at the 20-kb resolution. The PC1 value was used to indicate the A/B compartment status, where a positive PC1 value represented the A compartment (blue) and a negative value represented the B compartment (yellow). **c** Numbers of identified A/B compartments and TADs in FGSCs and other cells. **d** Average contact probability across the genome decreased as a function of the genomic distance

An overview of the intrachromosomal contact heat maps revealed that FGSCs had a distinct chromatin organization (Fig. 2a). We further examined the characteristics of the chromatin organization by analyzing the patterns of the compartment status and TADs in autosomes across cells, which avoided sex chromosome effects. The compartment status was classified as active (A) or inactive (B) (Suppl. Table S2). The result showed FGSCs had different A/B compartments compared with other cell types and the patterns of TADs or directional indexes (DIs) were almost the same for these cells (Fig. 2b). We counted the numbers of compartments and TADs in the cells and found that FGSCs had the lowest number of TADs and the number of compartments was similar to that in SSCs (Fig. 2c, Suppl. Table S3). We also calculated the average intrachromosomal contact probability of cells and found that the chromatin interaction frequency was decreased monotonically from 1 × 10^5^ to 1 × 10^8^ bp for FGSCs and the other cells (Fig. 2d). The contact probability curves were similar in the five cell types from 1 × 10^5^ to 1 × 10^8^ bp, but changes were observed at the long-distance genome as reported previously [47].

### Compartment status distinguishes FGSCs from iPSCs

**Fig. 3:**
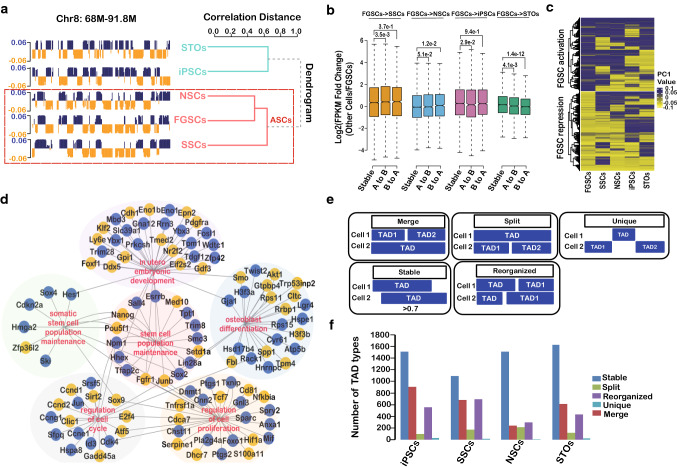
FGSCs exhibit a specific compartment status. **a** FGSCs were similar to other ASCs (left: example of PC1 score represented compartment status across five types of cells; right: hierarchical clustering of PC1 score). **b** Genes that changed compartment status (A to B or B to A) or those that remained the same (stable) compared with FGSCs (*p *value by Wilcoxon’s test). **c** *K*-means clustering (*k* = 2) of PC1 values of the genome that changed A/B compartment status in FGSCs. **d** Display of the GO enrichment of genes with changed compartment status in FGSCs. Genes were circled in blue color represented that they were located in A compartment, while orange color represented that they were located in B compartment. Each biological process was colored in the background. **f** Comparison of gene expression between TADs and TAD boundaries

Further systematic analysis of the compartment status across five cell types showed that FGSCs were more similar to NSCs and SSCs in terms of A/B compartments compared with iPSCs and STOs cells (Fig. 3a), suggesting that FGSCs were ASCs. Meanwhile, switching compartments of FGSCs accounted for about 40% proportion of compartments compared with iPSCs and STO cells, but a smaller proportion (30%) compared with SSCs and NSCs (Suppl. Figure S2A). These results suggested that FGSCs had a unique A/B compartment status that was more similar to other ASCs than iPSCs or STO cells. Furthermore, RNA-seq data showed genes were highly expressed in compartment A than in compartment B (Suppl. Figure S2B). The genes located in the switching compartment tended to be differentially expressed compared with the stable compartments (Fig. 3b), which indicated that the compartment status was correlated with gene expression. Unexpectedly, go enrichment of the genes located in A compartment of FGSCs showed there was no female or stem cells related pathways (Suppl. Figure S2C). Then we asked whether there was a pattern of compartment status could reflect FGSCs’ property. We used *K*-means method to cluster the PC1 value of compartment status of all cell types examined for characterizing FGSCs’ property. The result showed compartment status could be divided two cluster: FGSC activation compartments and FGSC repression compartments (Fig. 3c), indicating the compartment A and B only present in FGSCs. Gene Ontology (GO) analysis of the genes with changed compartment status in FGSCs showed that it was particularly associated with stem cell population maintenance and cell proliferation (Fig. 3d), suggesting that FGSC activation compartments were highly related with FGSCs’ biological property.

We next identified TADs in FGSCs using the direction index (DI) (Suppl. Table S3). Well-defined TADs were occupied in ~ 90% of the genome across cell types (Suppl. Figure S2D). According to the proportion of overlapping TADs compared with FGSCs, we classified the TADs into five types: stable, merged, split, reorganized, and unique (Fig. 3e). In which, two or more TADs in a stage fused into one TAD was defined as “merge”; one TAD divided into two or more TADs was defined as ‘split’; and a TAD was unique in cells was defined as ‘unique’. In addition to the merge, split, and unique TADs, a proportion of overlapping TADs > 0.70 was defined this as ‘stable’, and other TADs were defined as ‘reorganized’. We found most TADs were the stable type (Fig. 3f), which suggested that the TAD structure was highly stable across all five types of cells in accordance with a previous report [38]. Taken together, our data indicated that the compartment status was more characteristic than TADs and represented the features of cells across the five cell types.

### Active compartment-associating loops (aCALs) reveal the FGSC signature

**Fig. 4:**
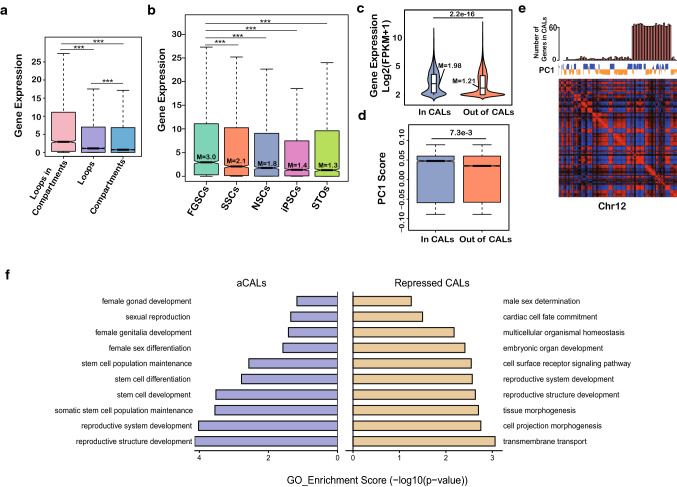
aCALs reveal the FGSC signature. **a** Expression of genes located in chromatin loops and compartments, only in chromatin loops, and only in compartments in FGSCs (*p *value by Wilcoxon’s test, *** represented *p* < 0.001). **b** Expression of genes located in chromatin loops and compartments across five type of cells (*M* means median; *p *value by Wilcoxon’s test, *** represented *p* < 0.001). **c** Gene expression in CALs and out of CALs in FGSCs (*M* means median; *p *value by Wilcoxon’s test). **d** PC1 values in CALs and out of CALs in FGSCs (*p *value by Wilcoxon’s test). **e** Example of chromosome 12 showed the Pearson’s correlation heat map, PC1 value, and gene density of CALs in FGSCs. **f** GO enrichment of genes in aCALs or repressed CALs

To systematically identify FGSC-specific functional chromatin loops using Juicer [19], we first identified 3332, 1064, 6031, 2941, and 5280 chromatin loops in FGSCs, SSCs, NSCs, iPSCs and STO cells, respectively (Suppl. Figure S3A,B). Next, we found that 3874, 1383, 5924, 3091, and 5031 genes in FGSCs, SSCs, NSCs, iPSCs and STO cells, respectively, were located in chromatin loops. Using a Venn diagram, we observed that 392 genes were shared across all cell types (cell type-shared loops) (Suppl. Figure S3C), which suggested that chromatin loops varied greatly among each cell type. To identify FGSC-specific chromatin loops, we further analyzed the data above. The results demonstrated that genes, which had formed chromatin loops located in compartments, had higher expression than in compartment boundaries and the compartment genes without chromatin loops forming in FGSCs (Fig. 4a). This suggested that the chromatin state-mediated compartment may contribute to gene regulation of chromatin loops. Further analysis of the expression of genes formed chromatin loops located in compartments showed they had highest expression in FGSCs compared with other type of cells (Fig. 4b), suggesting those specific loops were related with FGSCs. These specific loops were termed as compartment-associating loops (CALs). We found that expression of genes and the PC1 score in CALs were higher than those out of CALs in FGSCs (Fig. 4c, d). In addition, the CALs were not related to the chromatin length, gene density, or TAD density (Suppl. Figure S4). Subsequently, by measuring the number of genes located in CALs per 1Mbp, we observed that the distribution of gene number of CALs was not correlated with PC1 (Fig. 4e), which was not biased to A or B compartment status. Interestingly, most of CALs (about 96%) were located in TADs, but the expression of genes in CALs were higher than in TADs (Suppl. Figure S5A). When we divided the CALs into active (aCALs) and repressed CALs in accordance with the previously identified FGSC activation and repression compartment statuses, we found 1818 and 1262 genes located in aCALs and repressed CALs, respectively (Suppl. Figure S5B). By comparison with other cell types, we found aCALs were mostly specific to FGSCs (Suppl. Figure S5C). The genes located in aCALs were highly expressed in FGSCs compared with other cell types (Suppl. Figure S5D). GO analysis showed that the genes in aCALs were highly involved in female sex differentiation, female gonad development, and stem cell population maintenance, while genes in repressed CALs were related to male sex determination, embryonic organ development, and tissue morphogenesis (Fig. 4f). Those results indicated that the aCALs could be potentially the signature of FGSCs.

### CTCF is a potentially important factor in regulating aCALs of FGSCs

**Fig. 5:**
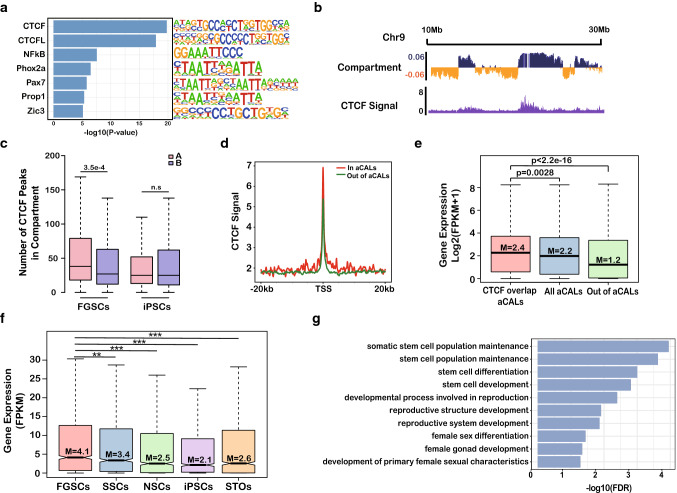
CTCF is a potential dominant factor in maintaining aCALs of FGSCs. **a** Motifs enriched in aCALs of FGSCs. **b** Snapshot of CTCF signals in A/B compartments in FGSCs. **c** Numbers of CTCF peaks in A/B compartments in FGSCs and iPSCs (*p *value by Wilcoxon’s test). **d** Enrichment of CTCF signals in the gene promoter of aCALs and out of aCALs. **e** Expression of genes shared by CTCF and aCALs, only in aCALs, and out of aCALs **(M** means median; *p *value by Wilcoxon’s test). **f** Expression of genes shared by CTCF and aCALs across five type of cells (*M* means median; *p *value by Wilcoxon’s test, ** represented *p* < 0.01, *** represented *p* < 0.001). **g** GO enrichment of genes shared by aCALs and CTCF

To further investigate the factor involved in regulation of the chromatin architecture in FGSCs, we performed motif analysis of aCALs. As a result, the top motifs within aCAL regions matched the CTCF motifs (Fig. 5a), which suggested that CTCF may be an important factor involved in regulation of aCALs in FGSCs. Next, we performed ChIP-seq of CTCF in FGSCs and found that ~ 86% of aCALs significantly overlapped with CTCF-binding sites (Suppl. Figure S6A, Fisher’s test *p* < 2.2e−16), which also suggested that CTCF plays a major role in the regulation of aCALs. Moreover, we observed that CTCF was enriched in the A compartment in FGSCs (Fig. 5b), which was in line with aCALs. By comparing the number of CTCF peaks between A and B compartments in FGSCs and iPSCs^48^, we found that CTCF was specifically enriched in the A compartment of FGSCs and not in iPSCs (Fig. 5c, Suppl. Figure S6B), which suggested specific enrichment of CTCF in the A compartment was FGSC and not in iPSCs. Furthermore, we found that genes located in aCALs had higher enrichment of CTCF than those out of aCALs (Fig. 5d). H3K4me3 and H3K27ac were also more enriched in the promoters of genes located in aCALs than in those out of aCALs (Suppl. Figure S6C), which suggested activation of more genes in aCALs. Subsequently, we identified 1566 overlapped genes between the CTCF-binding region and aCALs termed as CTCF-related aCAL genes. Indeed, RNA-seq results showed that CTCF-related aCAL genes had the highest expression among genes in and out of aCALs (Fig. 5e). Moreover, those CTCF-related aCALs genes had highest expression in FGSCs compared with other cell types, confirmed that those genes were related with FGSCs (Fig. 5f). GO analysis showed that the genes were involved in stem cell population maintenance, reproductive structure development and female gonad development (Fig. 5g). Taken together, our data indicated that CTCF was a potentially important factor in regulating aCALs of FGSCs and possibly involved in FGSC development.

### CTCF maintains the biological functions of FGSCs

**Fig. 6:**
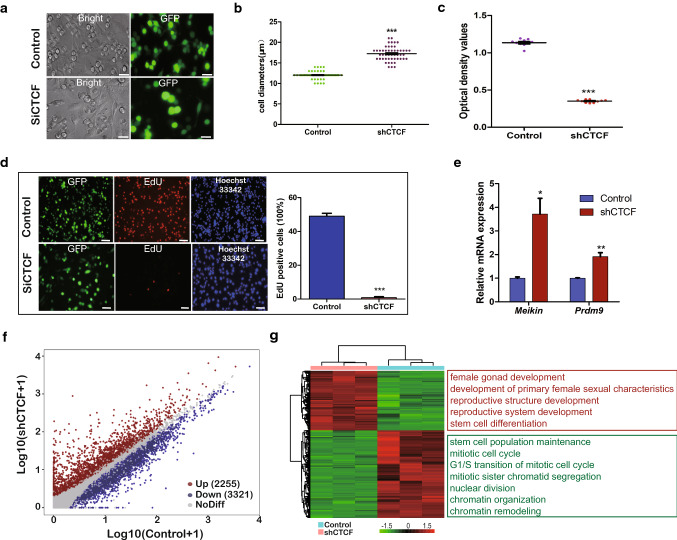
Effect of CTCF knockdown on the biological functions of FGSCs. **a** Morphology of shCTCF-treated FGSCs and the control. Scale bar, 25 µm. **b** Cell sizes in shCTCF and control groups. **c** CCK8 assays of shCTCF-treated FGSCs and the control. **d** EdU incorporation assays of shCTCF-treated FGSCs and the control. Scale bar, 50 µm. **e** qRT-PCR analysis of *Meikin* and *Prdm9* expression in shCTCF-treated FGSCs and the control. **f** Scatter plot of genes with significant differential expression in shCTCF-treated FGSCs and the control. **g** Heat map and GO enrichment showed the gene expression and biological processes in shCTCF-treated FGSCs and the control

To determine whether CTCF was implicated in the biological functions of FGSCs, we performed CTCF knockdown assays in FGSCs using short hairpin RNA (shRNA) technology (Suppl. Figure S7A). After CTCF knockdown in FGSCs, we observed that the size of FGSCs was obviously larger and the proliferative capacity of FGSCs was reduced significantly compared with the control (Fig. 6a–c). Furthermore, EdU incorporation assays demonstrated that the proportion of EdU-positive cells was significantly decreased in the shCTCF group compared with the control (Fig. 6d). To clarify the reason why cells viability had declined and the size of FGSCs was larger after shCTCF treatment, we measured the expression of meiotic kinetochore factor (Meikin) and PR domain zinc finger protein 9 (Prdm9), which are differentiation related-genes of germline stem cells, by qRT-PCR. Significant differences were found between the control and CTCF knockdown group (Fig. 6e). CTCF knockdown inhibited FGSC proliferation and induced FGSC differentiation. Next, RNA-seq data were generated from the CTCF knockdown group, which identified 2255 upregulated genes and 3321 downregulated genes (Fig. 6f). Notably, downregulated genes were enriched for stem cell population maintenance and mitotic sister chromatid segregation, while upregulated genes were related to stem cell differentiation and female gonad development (Fig. 6g). The expression of heat shock protein family A member 1B (Hspa1b), membrane anchored junction protein (Majin), and Notch receptor 2 (Notch2), which are differentiation or proliferation related-genes of germline stem cells, was measured by qRT-PCR to validate the RNA-seq data (Suppl. Figure S7B). Among them, 486 genes of CTCF-related aCALs showed significantly different expression between CTCF knockdown and control groups, in which 224 genes were up-regulated and 262 genes were down-regulated in CTCF knockdown group (Suppl. Figure S7C). GO analyses showed that they were related to stem cell development and female gonad development (Suppl. Figure S7D), which is consistent with our previous findings that CTCF-related aCAL genes may have a dominant role in FGSC development. In summary, these results indicated that CTCF might maintain the biological functions of FGSCs through regulation of chromatin organization.

### CTCF is required to maintain the high-order chromatin structure of FGSCs

**Fig. 7:**
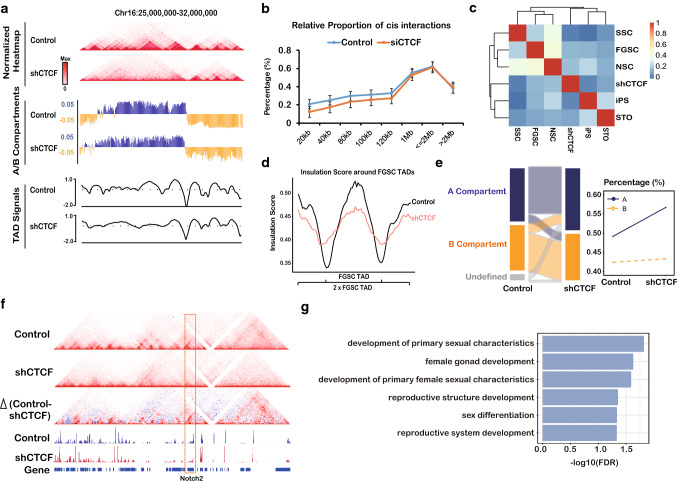
CTCF is required to maintain the high-order chromatin structure of FGSCs. **a** Examples of contact matrices of chromosome 16 in shCTCF-treated FGSCs and the control. Normalized Hi-C interaction heat maps of A/B compartments (A, blue; B, orange) and TAD signals in shCTCF-treated FGSCs and the control at the 20-kb resolution. **b** Relative proportions of *cis* interactions at different genome distances versus total paired loci in shCTCF-treated FGSCs and the control. **c** Pearson correlation of PC1 value between shCTCF-treated FGSCs and other type of cells. **d** Average insulation scores (ISs) in shCTCF-treated FGSCs and the control at TADs and nearby regions. **e** Alluvial plots of the switch of the compartment status in shCTCF-treated FGSCs and the control. **f** Notch2 expression was reduced in shCTCF-treated FGSCs, which did not form chromatin loops. **g** GO enrichment of differentially expressed genes of CTC-related aCALs, which did not form chromatin loops in shCTCF-treated FGSCs

To explore whether CTCF affected the high-order chromatin structure of FGSCs, a Hi-C experiment was implemented for the CTCF knockdown group (Suppl. Table S4). We observed the change of TADs and A/B compartments between the CTCF knockdown group and control (Fig. 7a). Globally, we calculated the proportion of interactions of less than 20, 40, 80, 100, or 120 kb versus total *cis*-interactions. The relative proportions of *cis*-short interactions were decreased in the CTCF knockdown group, which recovered with the distance of interactions (Fig. 7b, Suppl. Figure S8A), suggesting that CTCF was implicated in short interactions such as chromatin loops and TADs. The numbers of TADs in the CTCF knockdown group were reduced compared with the control (Suppl. Figure S8B). Furthermore, we found the high-order chromatin organization of CTCF knockdown group was closer to iPSCs and STOs (Fig. 7c), indicating that CTCF play an important role of maintaining the chromatin architecture of FGSCs.

To systematically evaluate the phenotype of CTCF in the high-order chromatin structure of FGSCs, we compared the Hi-C data between CTCF knockdown and control groups. TAD signals demonstrated that the CTCF knockdown group had significantly reduced strength of TADs compared with the control (Fig. 7d). A global change of the compartment status showed that the proportion of the A compartment was increased in the CTCF knockdown group (Fig. 7e). When compared with FGSC activation and repression compartments, we observed that some activation compartments had switched to repression in the CTCF knockdown group and vice versa (Suppl. Figure S8C). Genes located in the switching compartment tended to exhibit differential expression compared with the stable compartments (Suppl. Figure S8D). Importantly, CTCF knockdown dramatically reduced the number of chromatin loops, which had loss of about 95% of chromatin loops (Suppl. Figure S8E). GO analysis of genes in the eliminated chromatin loops showed they were also involved in female development and stem cell population maintenance (Suppl. Figure S8F). Moreover, 1707 genes of aCALs did not form chromatin loops in the CTCF knockdown group, which included 1480 genes of CTCF-related aCALs (Suppl. Figure S9A). Combined with RNA-seq data, we finally identified 466 genes of CTCF-related aCALs, in which 212 genes were up-regulated and 254 genes were down-regulated in CTCF knockdown group (Suppl. Figure S9B, C). Among them, *Notch2* showed higher expression than in the CTCF knockdown group in which it lost formation of the chromatin loop (Fig. 7f). These results indicated those genes were regulated by the formation of CTCF-mediated chromatin loops. Further analysis of enrichment of Gene Ontology showed that these genes were related to female development (Fig. 7g), which is consistent with previous findings. Taken together, these findings indicated that CTCF played an important role to maintain the high-order chromatin structure in FGSCs.

## Discussion

Stem cells, which include pluripotent stem cells (ESCs and iPSCs) and ASCs, have important implications in basic biology and regenerative medicine. Our previous studies have shown that FGSCs exist in postnatal ovarian tissues of mice, rats, humans, and pigs [2, 4–6, 49]. As a novel ASC, FGSC had potential applications in biotechnology and medicine. Therefore, their isolation from adult ovaries, long-term culture, and regulation of self-renewal and differentiation have gained a great deal of interest in stem cell biology and reproductive medicine [2, 4–6, 50–52]. Zou et al. showed that long-term-cultured FGSCs from adult mammals differentiate into oocytes after transplantation in vivo and play an important role in maintaining female fertility [2]. White et al. isolated human FGSCs from adult ovaries and maintained long-term cultures of these cells [4]. Wu et al. characterized and traced the development of transplanted FGSCs of long-term culture in vivo [3]. By integrative epigenomic analysis, unique epigenetic signatures involved in unipotency of FGSCs have been revealed [32]. However, the regulatory mechanism of the FGSC signature based on the high-order chromatin structure remains to be explored.

To identify the chromosome structure characteristics of FGSCs, we compared FGSCs with pluripotent stem cells (iPSCs), ASCs (SSCs and NSCs), and somatic cells (STO cells) by Hi-C technology. The results revealed that FGSCs had a distinct high-order genome structure in terms of the A/B compartment status, chromatin loops, and TADs. For further characterization, we identified FGSC-specific activated and repressed compartment regions, and identified some genes that were highly related to the switch of the FGSC compartment status. These genes were related to stem cell maintenance and differentiation pathways, which strongly supports the role of FGSCs as ASCs with some shared characteristics with SSCs and NSCs. This suggests that the compartment status was highly specific to the cells. However, the further analysis of TADs showed that they were stable across stem cells, while chromatin loops analysis demonstrated that there were less shared chromatin loops, which indicated that chromatin loops may be another feature of the 3D chromatin structure. Hence, we propose that the regulation of the 3D chromatin architecture is not at a single level. By the combination of the distinct compartments and chromatin loops in FGSCs, we finally identified aCALs and repressed CALs in FGSCs. The results of GO analysis showed that the genes of aCALs were involved in female development and stem cell development, which suggested that these aCALs were features of FGSCs and consistent with FGSC biology. Our data indicated that the characteristics of FGSCs were regulated by cooperation through multiple levels of the high-order chromatin structure, such as the compartment status and chromatin loops, which has revealed the features of the 3D chromatin architecture.

By further exploring these specific chromatin loops, CTCF was identified as an important factor to regulate these features. Thus, we knocked down CTCF and found that CTCF knockdown inhibited FGSC proliferation and induced differentiation. Combined with RNA-seq and Hi-C results, we also found that the CTCF knockdown group lost most of the specific chromatin loops in which genes that did not form chromatin loops were enriched in female development. These findings not only revealed that CTCF is involved in maintaining the properties of FGSCs through regulating the high-order chromatin structure, but also suggested that FGSC development in vitro can be used to study female germline cell development.

Previous researches reported CTCF shaped the chromatin structure accompanied with cohesin complex [20, 53–55]. It raised a question whether cohesion complex is related with the maintenance of FGSCs. Several lines of evidence showed that cohesion complex was involved in the development of germline cells and stem cells. First, it is reported that cohesion complex played important role in meiosis process [56–58], which disruption of cohesion complex led to sterile in mouse. Secondly, cohesion complex is necessary for the maintenance of self-renewal genes in stem cells [59], which depletion of cohesin led to the abolishment of enhancer–promoter stabilization of self-renewal genes. Moreover, it is reported that cohesion complex not only maintain the self-renewal, but also can block the differentiation in epidermal progenitor cells and intestinal stem cells [60, 61]. Last but most important, we found the expression of subunit of cohesin complex (SMC1a, SMC3, Rad21) was down-regulated in knock down CTCF FGSCs, suggesting that there was relationship between CTCF and cohesin complex. These findings suggested that cohesin complex is a potential factor to maintain the FGSCs in conjunction with CTCF.

## Conclusion

In conclusion, we present a comprehensive overview of the chromatin organization in FGSCs to create a rich resource for genome-wide maps. Our findings revealed that the chromatin architecture of FGSCs included a unique compartment status and chromatin loops, especially aCALs, which may contribute to their cell type-specific gene regulation. Furthermore, CTCF was identified to play an important role in maintaining the biological functions of FGSCs by regulating the chromatin organization. These data provide a valuable resource for future studies of the features of chromatin organization in mammalian stem cells and further understanding of the fundamental features of FGSCs.

### Supplementary Information

Below is the link to the electronic supplementary material.

Supplementary file1 (PDF 1172 KB)

Supplementary file2 (XLSX 12 KB)

Supplementary file3 (XLSX 107 KB)

Supplementary file4 (XLSX 294 KB)

Supplementary file5 (XLSX 46 KB)

Supplementary file6 (XLSX 12 KB)

## Declarations

### Availability of data and material

The accession number for the expression and sequencing data reported in this paper are in GEO: GSE126014, GEO: GSE137771 and GEO: GSE175730. All customer codes are publicly available via GitHub (https://github.com/skytguuu/Geng_2019). All other data contained in the article and in its supplementary information are available upon request.
