# Supplementary material for: Integrative analysis of the 3D genome structure reveals that CTCF maintains the properties of mouse female germline stem cells
Source: Cell Mol Life Sci. 2022 Jan 3;79(1):22. doi: 10.1007/s00018-021-04107-y (PMC8724064; doi:10.1007/s00018-021-04107-y)
Supplement: Supplementary file 1 — Supplementary file1 (PDF 1172 KB) [file 18_2021_4107_MOESM1_ESM.pdf]

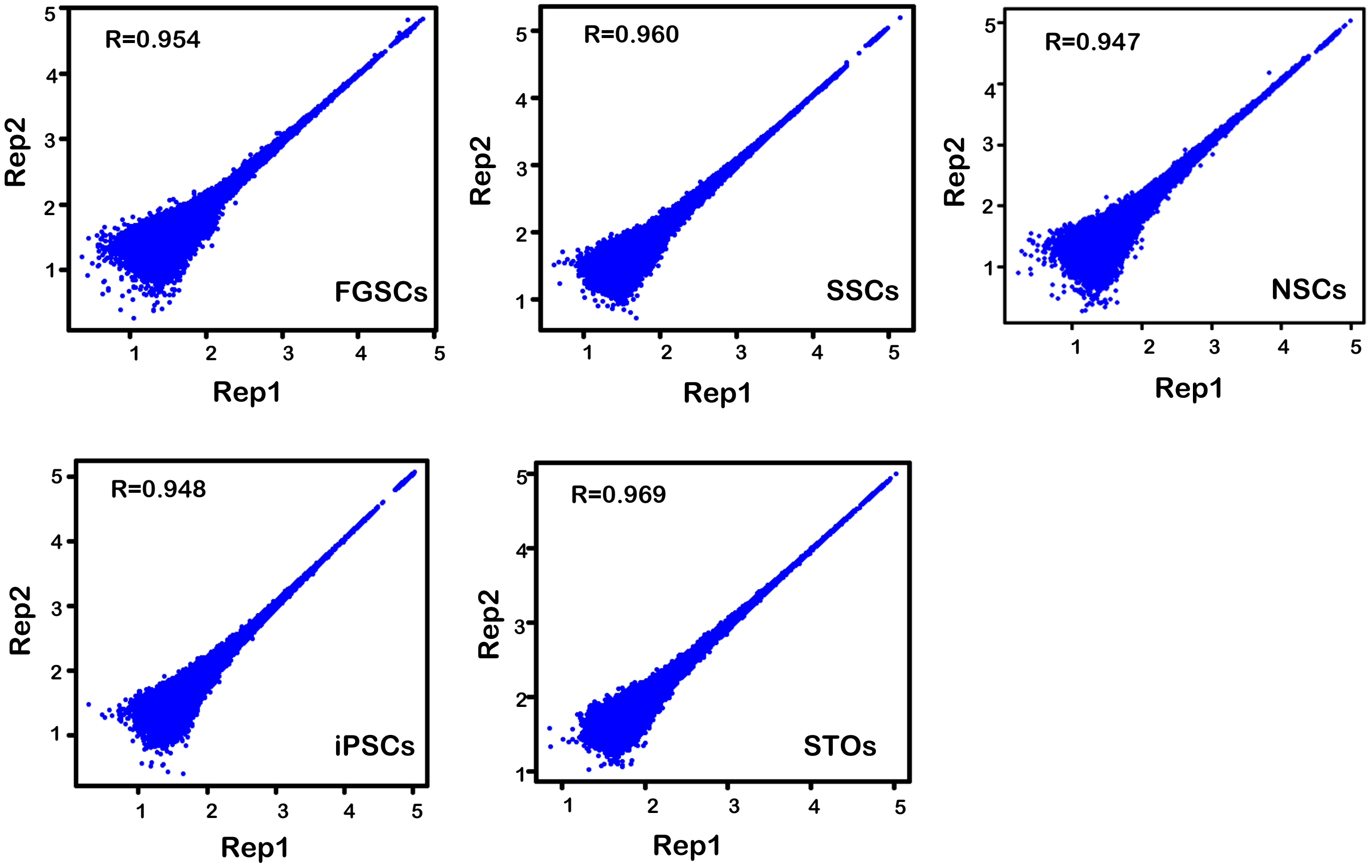

Figure S1. Validation of Hi-C data quality.

The correlation between Hi-C replicates for each cell type according to the normalized interaction frequency at 400-kb resolution. R indicates Pearson's correlation coefficient.

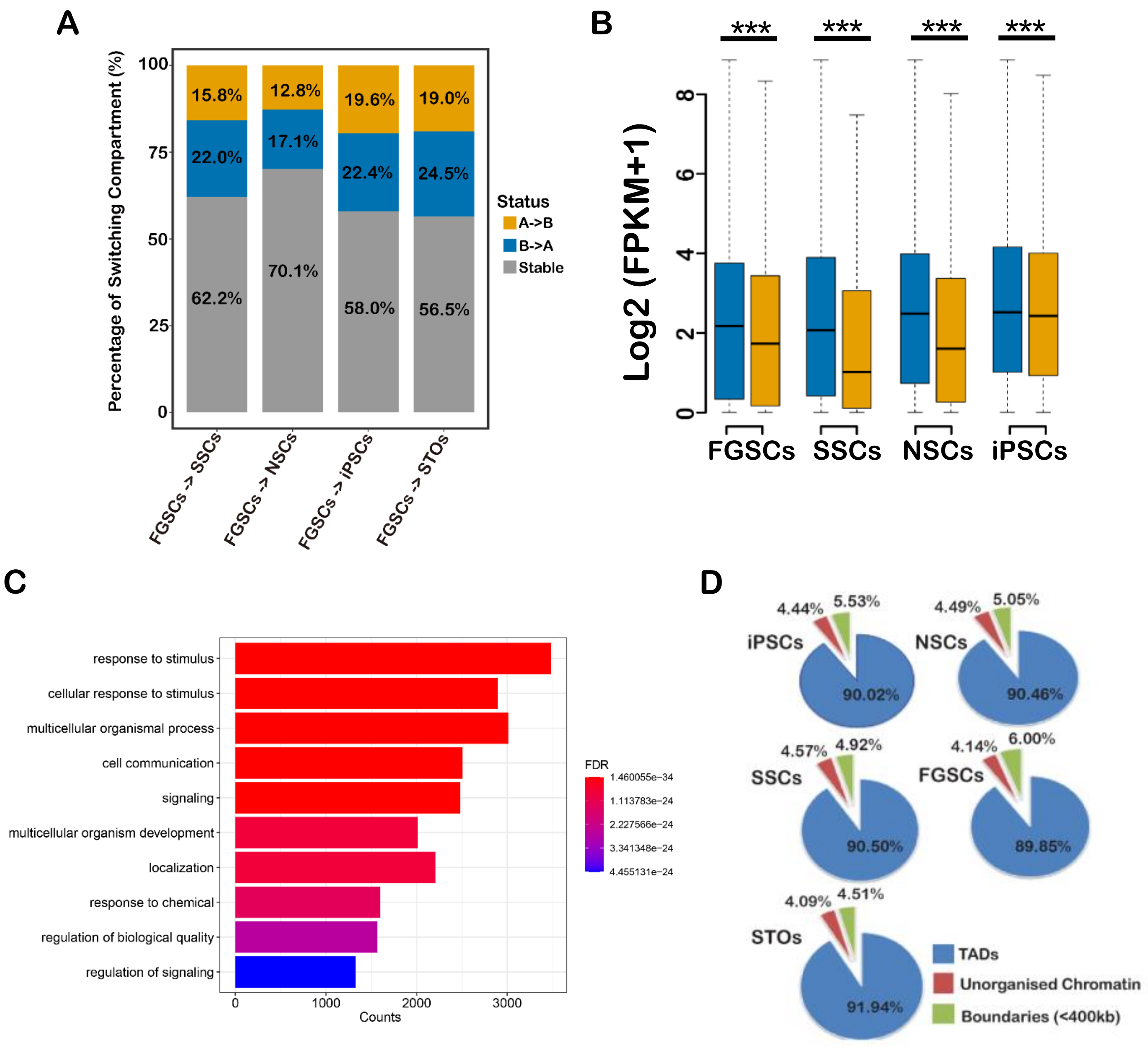

Figure S2. **The switch of A/B compartment correlated with gene expression.**

(A) Percentage of switching A/B compartment status in FGSCs, compared to other types of cells.

(B) Expression of genes with A or B compartment status across stem cells (*: $p<0.05$; **: $p<0.01$; ***: $p<0.001$; p value by Wilcoxon's test).

(C) Go enrichment of genes located in A compartment of FGSCs.

(D) Percentages of TADs and TAD boundaries in the genome across each type of cells.

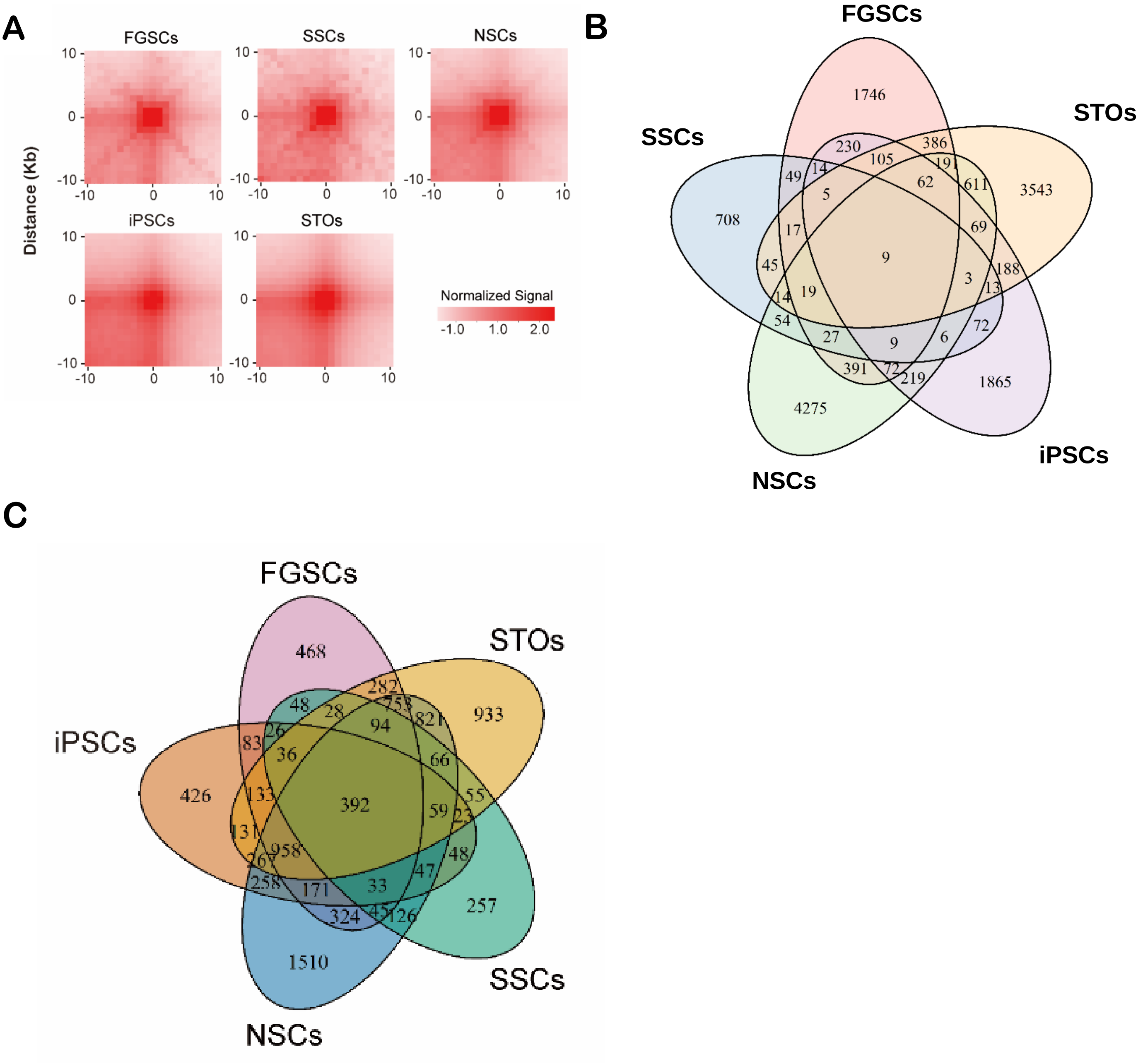

Figure S3. **Chromatin loops across each type of cells.**

(A) APA score of Chromatin loops in FGSCs, SSCs, NSCs, iPSCs and STOs.

(B) Venn diagram showing that a few loops were shared across all cell types

(C) Venn diagram showing that a few genes were shared across all cell types.

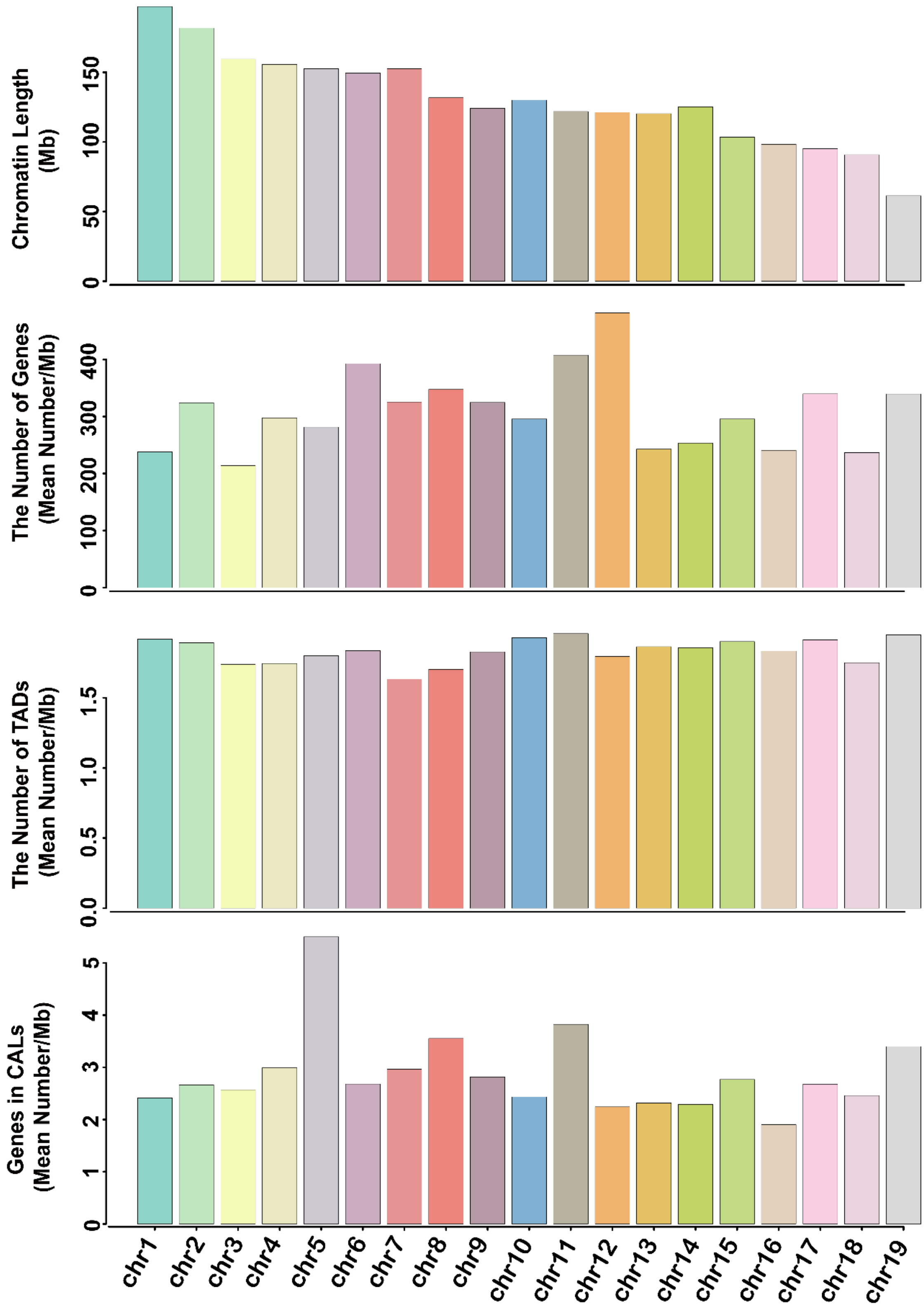

Figure S4. **The number of genes in CALs in each chromosome.**

Chromatin length, gene density, TAD density and CALs' gene density were shown.

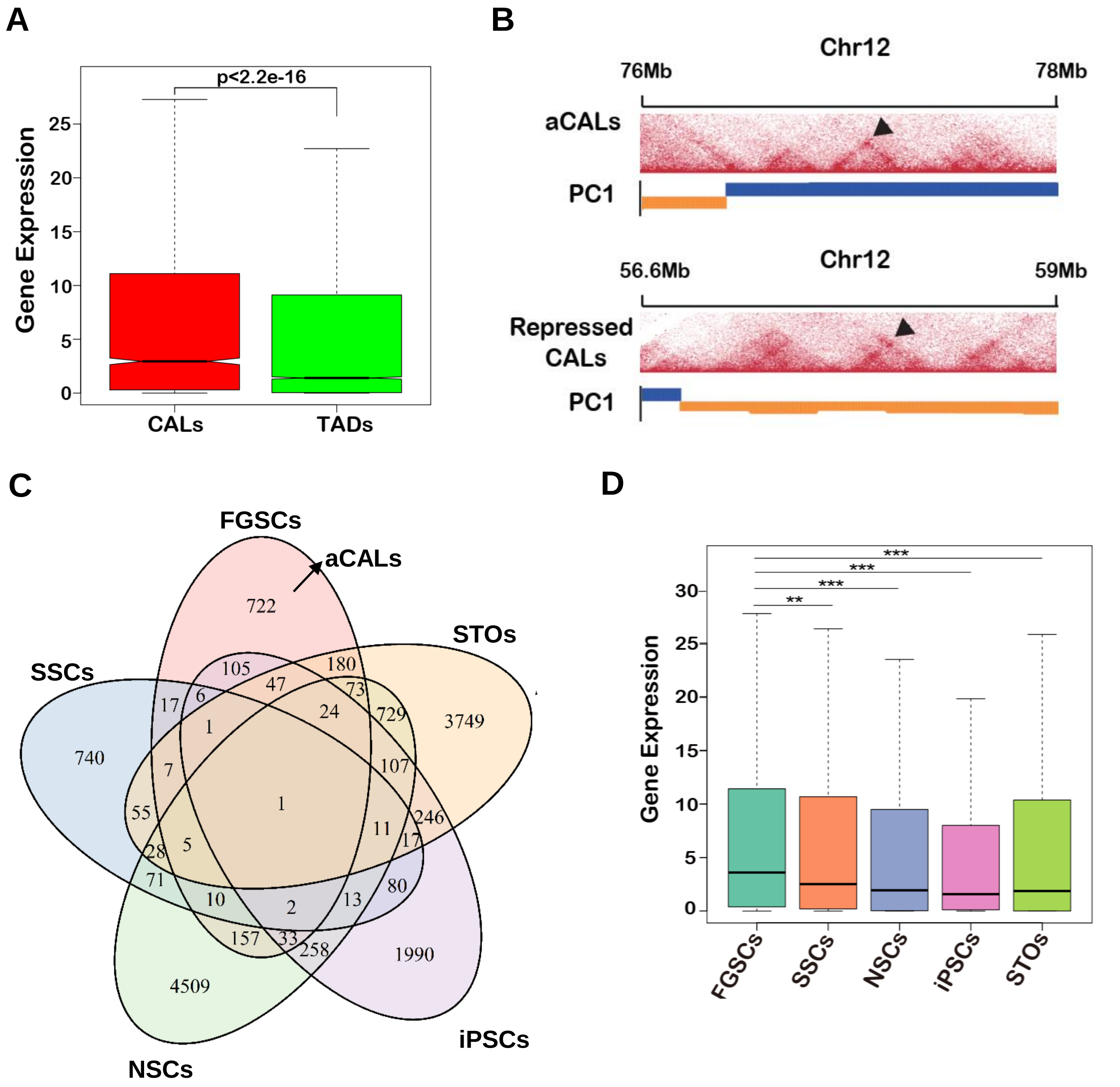

Figure S5. **aCALs were the feature of chromatin organization to FGSCs.**

(A) Boxplot showed the expression of genes located in CALs and TADs (p value by Wilcoxon's test).

(B) CALs were divided into active and repressed CALs in accordance with the PC1 value.

(C) Venn diagram showed that most aCALs were specific to FGSCs compared with other type of cells.

(D) Expression of genes of aCALs across five type of cells. (P-value by Wilcoxon's test, ** represented p<0.01, *** represented p<0.001)

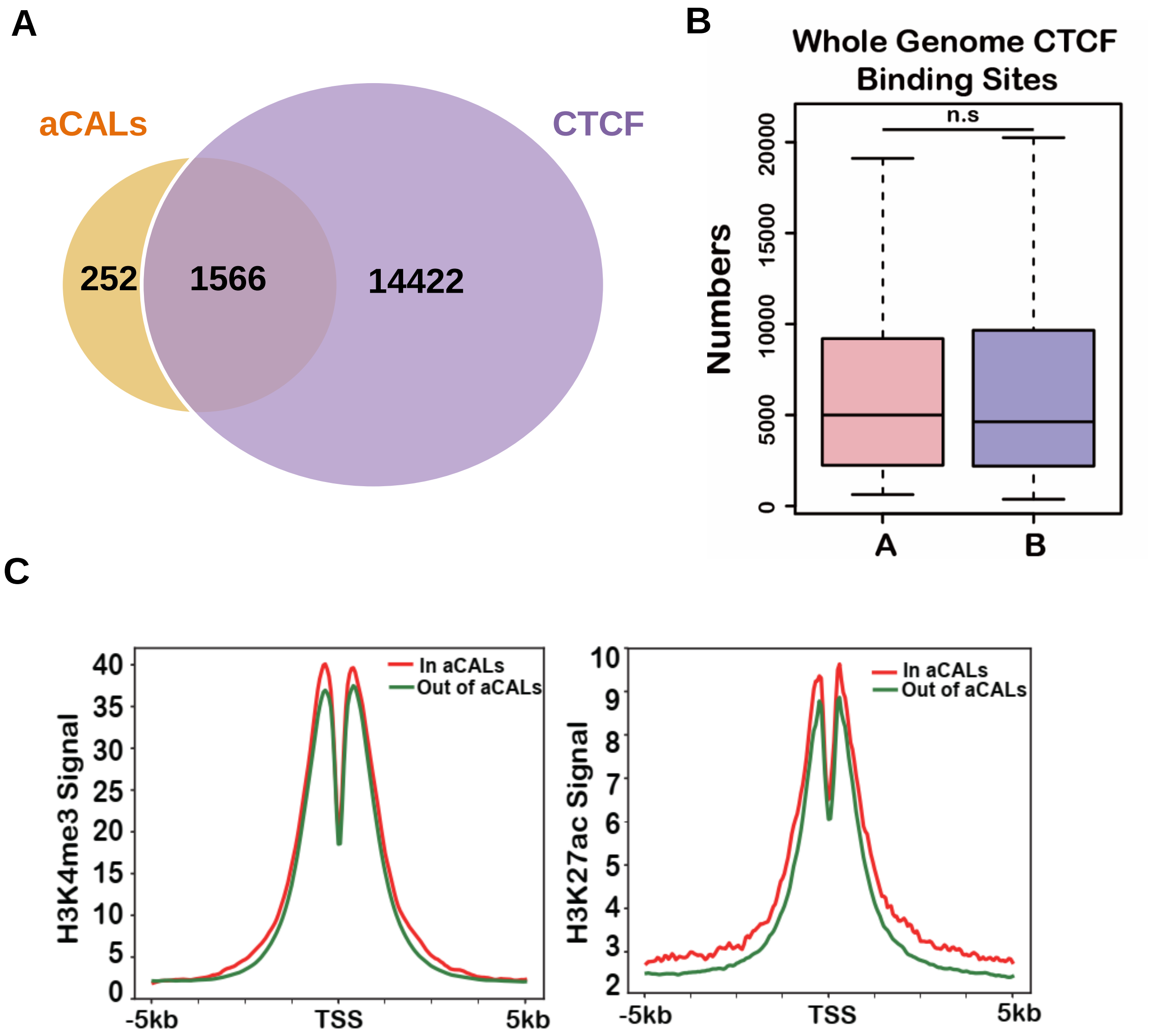

Figure S6. CTCF is a potential key factor for aCALs.

(A) Venn diagram showed that most genes of aCALs were shared by CTCF.

(B) The number of CTCF peaks in A/B compartments in the whole genome of mouse showed the CTCF was no biased to A or B compartment.

(C) Enrichment of H3K4me3 and H3K27ac signals in the gene promoter of aCALs and out of aCALs.

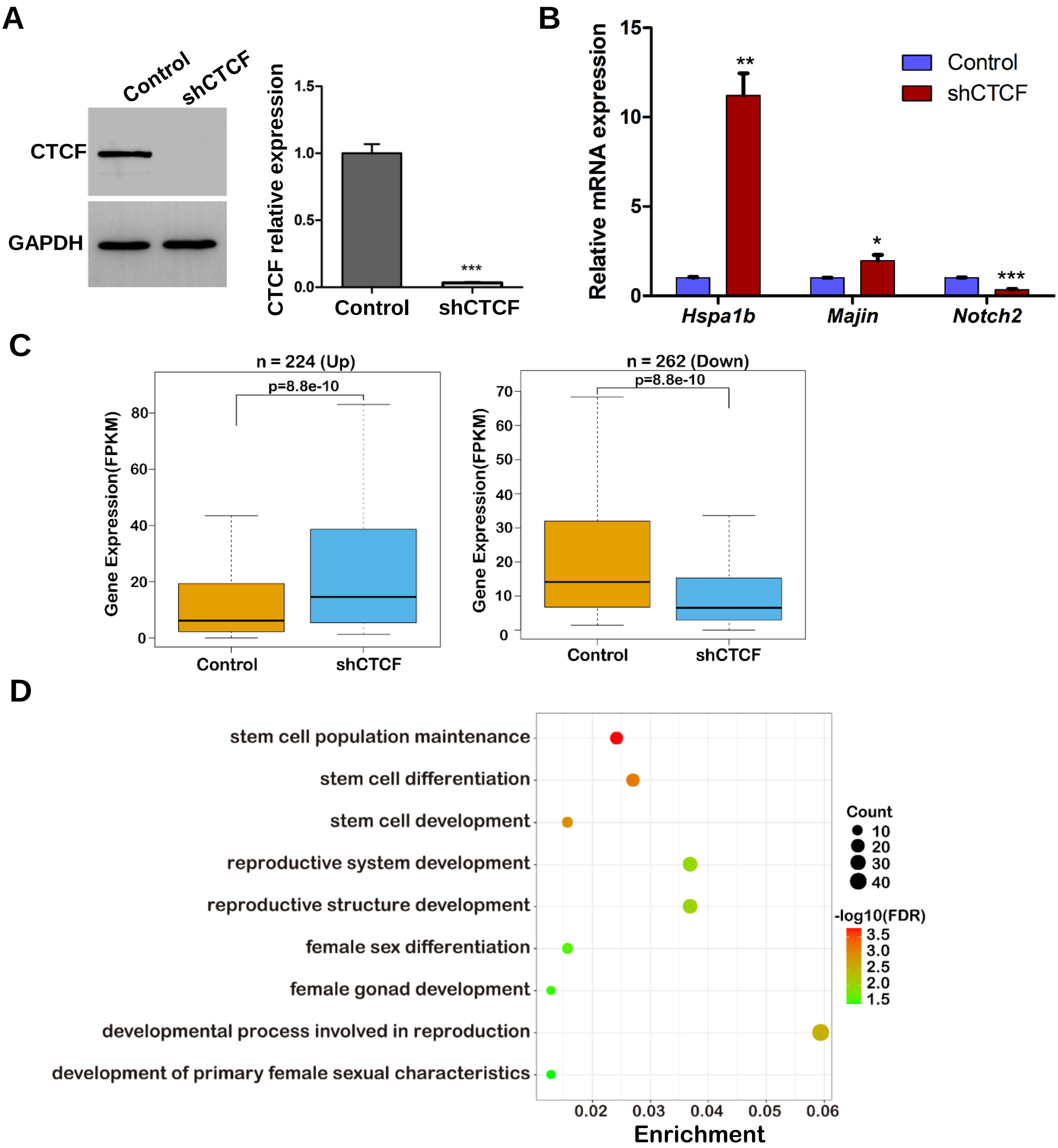

Figure S7. The expression of CTCF in FGSCs or shRNA group .

(A) Western blot showed the knockdown efficiency of CTCF in FGSCs.

(B) qRT-PCR analysis of the expression of Hspa1b, Majin and Notch2 in shCTCF FGSCs and control.

(C) Boxplot showed the up or down regulated genes of CTCF-related aCALs. (P-value by Wilcoxon's test)

(D) Functional enrichment of different expressed CTCF related aCALs genes.

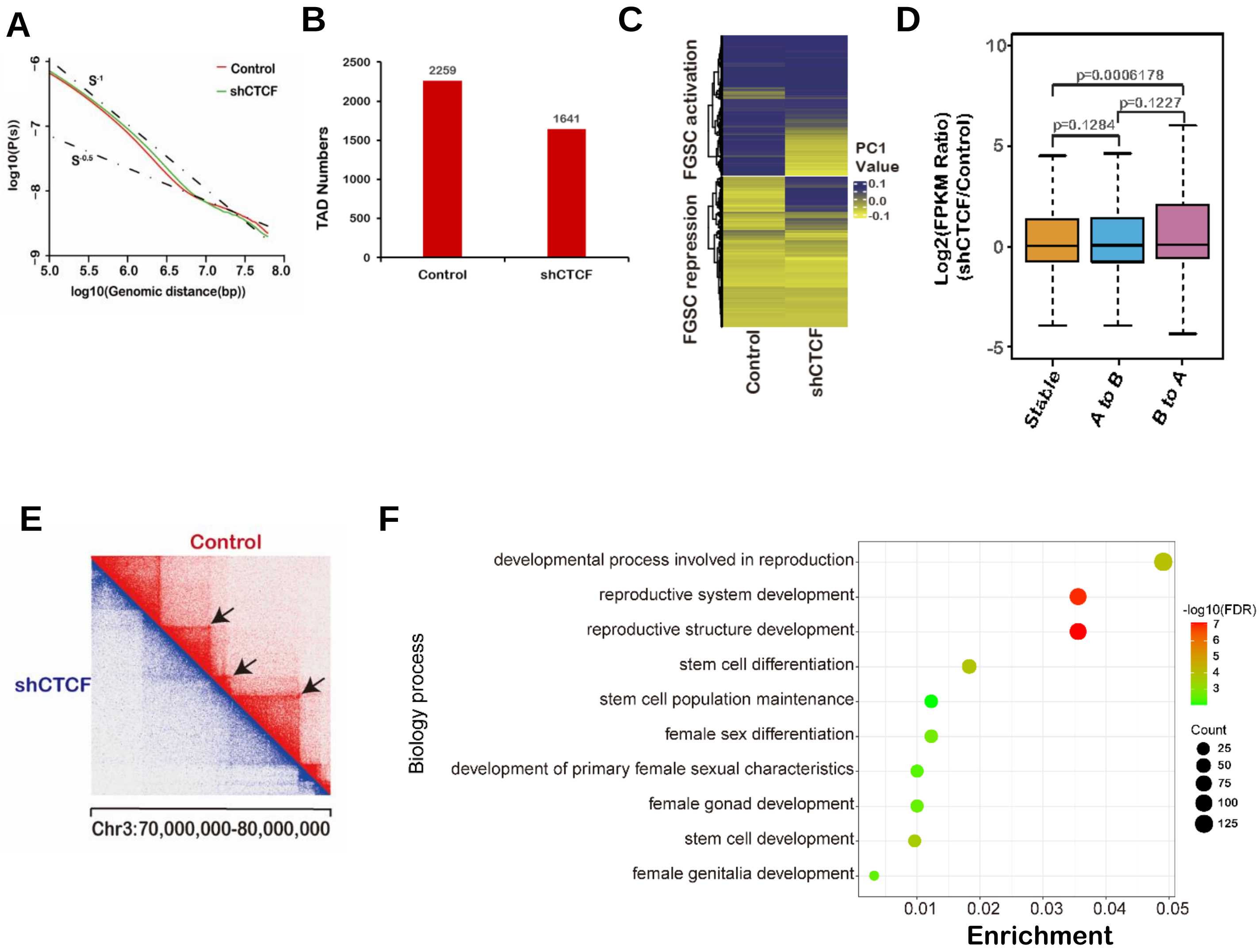

Figure S8. Hi-C analysis of the shCTCF group in FGSCs.

(A) Average contact probability across the genome was decreased as a function of the genomic distance in shCTCF and control groups.

(B) Numbers of TADs in shCTCF and control groups.

(C) Heatmap of PC1 values in shCTCF-treated FGSCs and the control.

(D) Genes that changed compartment status (A to B or B to A) or those that remained the same (stable) compared with the control (P-value by Wilcoxon's test).

(E) CTCF loss eliminated chromatin loops in the shCTCF group.

(F) Functional enrichment of genes that did not form chromatin loops in the shCTCF group.

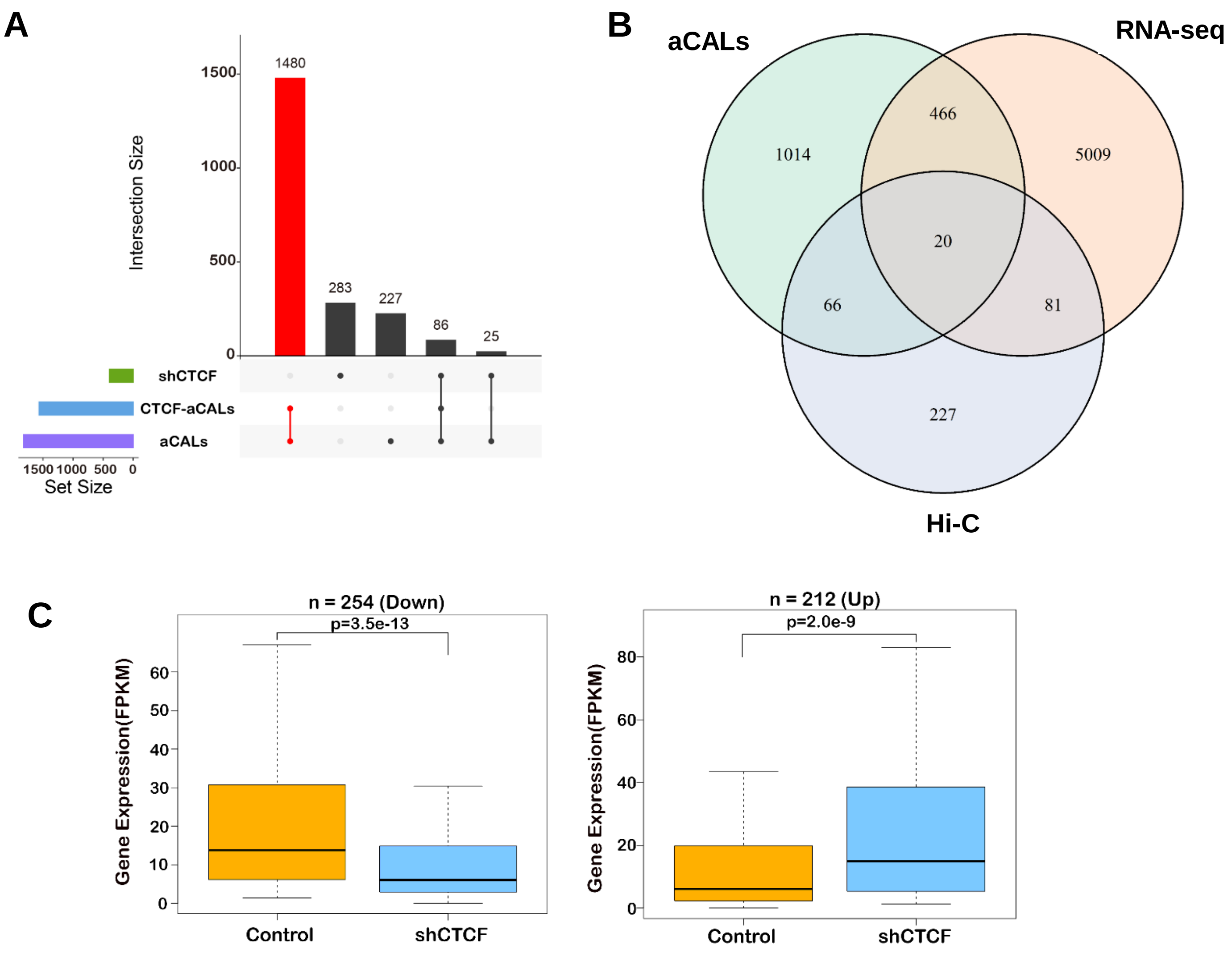

Figure S9. **CTCF-mediated chromatin loops in FGSCs.**

(A) Venn diagram showed that most genes of aCALs lost to form chromatin loops were CTCF-related aCALs.

(B) Venn diagram showed that the different expressed genes of CTCF-aCALs were failed to form the chromatin (RNA-seq represented different expressed genes in shCTCF; Hi-C represented the genes formed chromatin loops in shCTCF).

(C) Boxplot showed the lost of chromatin loops of genes of CTCF-related aCALs were up or down regulated in shCTCF group. (P-value by Wilcoxon's test).
